# Anatomic Landmarks for the First Dorsal Compartment

**Published:** 2009-06-02

**Authors:** Ron Hazani, Nitin J. Engineer, Damon Cooney, Bradon J. Wilhelmi

**Affiliations:** Division of Plastic and Reconstructive Surgery, University of Louisville School of Medicine, Louisville, KY

## Abstract

**Objective:** Knowledge of anatomic landmarks for the first dorsal compartment can assist clinicians with management of de Quervain's disease. The radial styloid, the scaphoid tubercle, and Lister's tubercle can be used as superficial landmarks for the first dorsal compartment. **Methods:** Thirty-two cadaveric wrists were dissected, and measurements were taken from the predetermined landmarks to the extensor retinaculum. The compartments were also inspected for variability of the abductor pollicis longus tendon and intracompartmental septations. **Results:** The average length of the extensor retinaculum from its proximal to distal extent measured approximately 2.2 cm. The distal aspect of the radial styloid was 0.3 cm distal to the distal aspect of the extensor retinaculum, and the distance between the distal aspect of the extensor retinaculum and the APL-Lister's-Scaphoid juncture was approximately 0.5 cm. A separate compartment for the extensor pollicis brevis was noted in 35% of the specimens. The abductor pollicis longus tendon demonstrated great variability with 1, 2, 3, or 4 slips in 9%, 30%, 43%, or 26% of the specimens, respectively. **Conclusion:** The superficial bony prominences of the radial wrist can be used reliably as anatomic landmarks for the first dorsal compartment.

De Quervain's disease is a common cause of wrist pain and disability. Tendon entrapment of the first dorsal compartment can be managed with conservative or surgical modalities.^1^ Treatment strategies include immobilization, corticosteroid injections, and operative release. Successful nonoperative management with corticosteroid injections has been demonstrated repeatedly in 50% to 80% of patients,^2^–^4^ particularly in acute cases.^5^ Maintaining a high level of success with corticosteroid injections may depend on our knowledge of the first dorsal compartment for precise needle placement. In this study, we propose the use of identifiable bony landmarks in the radial aspect of the wrist for proper location of the first dorsal compartment.

Numerous anatomic and surgical studies have shown great variability in tendon structure and organization of the first dorsal compartment.^6^–^10^ Septations within the osteofibrous tunnel and slip multiplicity of the abductor pollicis longus (APL) have all been raised as possible causes of de Quervain's tenosynovitis,^11^ or causes for failed steroid injections.^6^ Despite the multitude of anatomic studies, none have addressed the need for clear anatomic landmarks required for successful injection. We report our measurements of bony landmarks for the first dorsal compartment and their application to the nonoperative management of de Quervain's disease.

## METHODS

**Figure 1:**
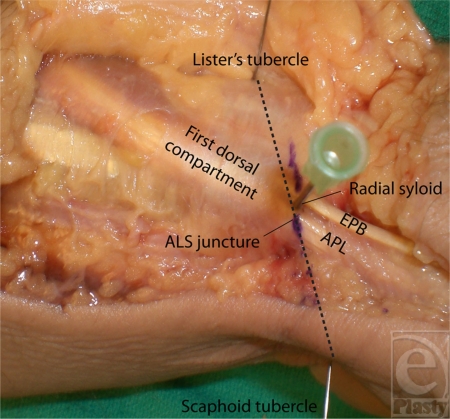
Anatomic landmarks for the first dorsal compartment. APL, abductor pollicis longus; ALS, APL-Lister's-Scaphoid; EPB, extensor pollicis brevis.

Thirty-two cadaveric wrists were dissected with the aid of loupe magnification. A longitudinal incision along the radial aspect of the wrist facilitated exposure of the first dorsal compartment, the radial styloid, and Lister's tubercle. The wrist and thumb were placed in a neutral position. Measurements of the distance between the distal aspect of the radial styloid and the distal edge of the extensor retinaculum were taken. Additional measurements were collected after proper identification of Lister's tubercle and the scaphoid tubercle. These bony prominences of the wrist (Lister's tubercle and scaphoid tubercle) are along a diagonal line which intersects the longitudinal course of the APL tendon. We marked the point of intersection as the APL-Lister's-Scaphoid (ALS) juncture. The distance from the distal aspect of the first dorsal compartment to the ALS juncture was recorded (Fig 1).

The first dorsal compartment length was measured and then opened for inspection of its content. Care was taken not to disrupt atypical intracompartmental septations. The extensor pollicis brevis (EPB) and APL were inspected for slip multiplicity. Our statistical analysis is reported in terms of average length and standard deviation of the various measurements.

## RESULTS

The average length of the extensor retinaculum from its proximal to distal extent measured 2.19 ± 0.37 cm. The distal aspect of the radial styloid was 0.32 ± 0.57 cm distal to the distal aspect of the extensor retinaculum. Therefore, the proximal edge of the extensor retinaculum is approximately 2.51 cm proximal to the radial styloid. Similarly, the distance between the distal aspect of the extensor retinaculum and the ALS juncture was 0.46 ± 0.36 cm.

**Figure 2:**
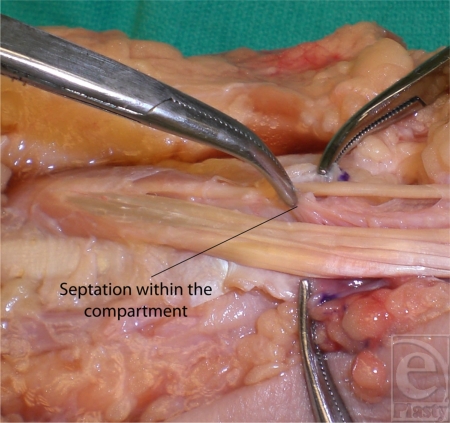
Intracompartmental septation within the first dorsal compartment for the extensor pollicis brevis tendon.

**Figure 3:**
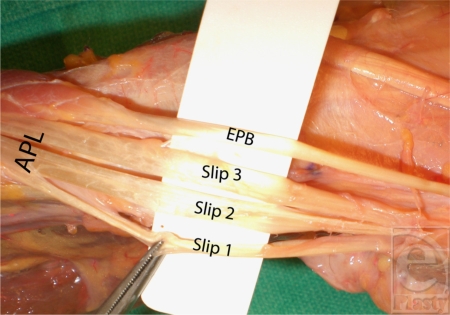
Slip multiplicity of the abductor pollicis longus tendon. APL, abductor pollicis longus; EPB, extensor pollicis brevis.

When the first dorsal compartment was opened for inspection, a separate compartment for the EPB was noted in 34.7% of the specimens (Fig 2). The EPB always had 1 slip. In contrast, the APL tendon demonstrated great variability with 1, 2, 3, or 4 slips in 9%, 30%, 43%, or 26% of the specimens, respectively (Fig 3).

## DISCUSSION

**Figure 4:**
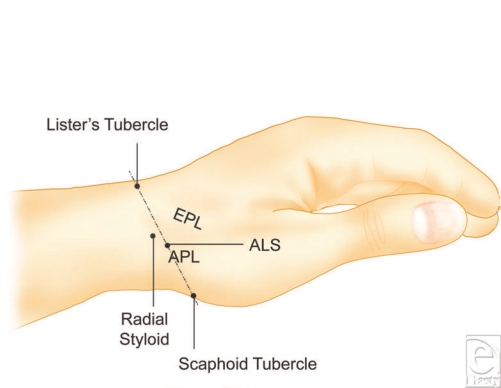
Illustration of the anatomic landmarks used for location of the first dorsal compartment. APL, abductor pollicis longus; EPL, extensor pollicis longus; ALS, APL-Lister's-Scaphoid.

In the majority of patients, the distal aspect of the radial styloid can be easily palpated and used for preinjection marking. When large body habitus interferes with accurate location of the radial styloid, other bony prominences can be considered. We use the ALS juncture as an alternative method for locating the distal edge of the first dorsal compartment. Lister's tubercle and the scaphoid tubercle are easily palpable in many patients, and the APL is seen with full abduction of the thumb (Fig 4). Both methods allow clinicians to accurately predict the location of the first dorsal compartment prior to needle insertion. After skin marking, the needle can be introduced 2.51 cm proximal to the radial styloid and angled distally at 45 degrees to the longitudinal axis of the radial aspect of the forearm.

A likely cause of incomplete resolution of tenosynovitis is anatomical inconsistency.^6^ It is suspected that the first dorsal compartment of the wrist is probably the site of most numerous variations in tendon structure and organization in the upper limb.^10^ A thorough understanding of the common anatomical variations within the first extensor compartment is also important for the operative treatment of de Quervain's tenosynovitis.

Previous anatomic studies of the first dorsal compartment reveal a significant number of tendon variations.^6^–^10^ The standard arrangement of tendons refers to a single APL inserting into the base of the metacarpal of the thumb or into the trapezium while the EPB inserts into the proximal phalanx.^11^ Aktan^10^ reported that in 85.4% of cadaveric wrists, the number of tendon slips differed from the standard. Septation was found in 10% of the wrists. The septum was between the APL and the EPB tendons. No third deep tunnel was found in all wrists. Kulthanan^9^ demonstrated supernumerary APL tendons in 89%, duplicated EPB tendons in 2%, and septation in 37% of cadaveric wrists. Our experience confirms the variability noted in previous studies, with supernumerary APL tendons in 89% and a single intracompartmental septation in 35% of the specimens. In contrast, no variability was present with regard to the EPB tendon. Given the anatomic variability demonstrated in this study and others,^6^–^11^ we share the belief that septations within the osteofibrous tunnel and slip multiplicity of the APL are possible causes of de Quervain's tenosynovitis^11^ or causes for failed steroid injections.^6^

## CONCLUSION

Knowledge of anatomic landmarks for the first dorsal compartment could significantly improve the approach and success rate of corticosteroid injections for de Quervain's disease. We found the radial styloid, Lister's tubercle, and the scaphoid tubercle as reliable landmarks for the location of the first dorsal compartment.

## ACKNOWLEDGMENT

We thank Margaret Abby and Mary B. Carter, MD, PhD, for their assistance with our statistical analysis.
